# Improvement on Permeability of Cyclic Peptide/Peptidomimetic: Backbone *N*-Methylation as A Useful Tool

**DOI:** 10.3390/md19060311

**Published:** 2021-05-27

**Authors:** Yang Li, Wang Li, Zhengshuang Xu

**Affiliations:** 1 State Key Laboratory of Chemical Oncogenomics and Laboratory of Chemical Genomics, Engineering Laboratory for Chiral Drug Synthesis, Peking University Shenzhen Graduate School, Shenzhen 518055, China; liwang18435165641@163.com; 2 Shenzhen Bay Laboratory, Shenzhen 518055, China

**Keywords:** backbone *N*-Methylation, cyclic peptide, cyclic peptidomimetic, permeability

## Abstract

Peptides have a three-dimensional configuration that can adopt particular conformations for binding to proteins, which are well suited to interact with larger contact surface areas on target proteins. However, low cell permeability is a major challenge in the development of peptide-related drugs. In recent years, backbone *N*-methylation has been a useful tool for manipulating the permeability of cyclic peptides/peptidomimetics. Backbone *N*-methylation permits the adjustment of molecule’s conformational space. Several pathways are involved in the drug absorption pathway; the relative importance of each *N*-methylation to total permeation is likely to differ with intrinsic properties of cyclic peptide/peptidomimetic. Recent studies on the permeability of cyclic peptides/peptidomimetics using the backbone *N*-methylation strategy and synthetic methodologies will be presented in this review.

## 1. Introduction

The development of drugs against protein-protein interactions (PPIs) is challenging for small molecules [1,2]. It is a rule of thumb that, for PPIs, small molecules are not able to bind to the large and flat binding sites with high affinity, as monoclonal antibodies do. On the other hand, monoclonal antibodies with high affinity to proteins are generally restricted to extracellular targets due to their limited cell membrane permeability. It is not easy for biomolecule drugs to cross biological membranes and take action on intracellular PPI targets. Specifically, peptide drugs, especially cyclic peptides and peptidomimetics display more advantages than both small molecule drugs and monoclonal antibodies on targeting PPIs, attributing to their appropriate molecular size and tunable molecular properties [3]. The size of peptides is generally larger than that of conventional small molecules, attributing them an antibody-like affinity for binding to flat PPI interfaces with relatively smaller molecule weight comparing to antibodies. Additionally, the procurability of structural diversity for peptides, using well-established synthetic chemistry methods, also laid the foundation for thorough investigations into how to improve their permeability [4,5,6,7]. With high molecular structural diversity, peptide and peptidomimetic skeleton offer a particularly high potential to achieve enormous structural variations by simply replacement or modifications of amino acid fragments (e.g., *N*-methylation). The synthesis of peptides and peptidomimetics have been well documented [8] and it is easy to generate a large number of molecules at low costs [9,10].

## 2. Cyclic Peptides in Drug Development

### 2.1. Peptides as Drugs

Targeting PPIs is an attractive therapeutic strategy for many kinds of diseases as PPIs are central to all biological processes and are often dysregulated in diseases [11]. In recent years, the development of PPI inhibitors has attracted a lot of attention. However, unlike the well-defined hydrophobic binding pockets for small molecule drugs, the interfaces of PPIs are usually dynamic and typically flat, which have limited the application of conventional small molecule on PPI targets. On the other hand, although biomolecules, such as monoclonal antibodies, show good binding affinity to PPIs, they lack cell permeability. Poor cellular permeability has greatly retarded the ability of biomolecules to be developed into drugs that target intracellular proteins. Compared to small-molecule drugs and biomolecule drugs, the chemical space of peptides, especially cyclic peptides and peptidomimetics, have greater potentials and are worthy of exploration. Peptides have three-dimensional configurations that tend to be disk-shaped or spherical, which can engage larger surface areas on targeted proteins. Peptides have higher targeting specificity and affinity, thus are potential PPI modulators. Peptides, especially cyclic peptides, retain several attributes of small molecules, such as stability and lower risk of an immune response, which makes them a good resource for PPI targeted drug development. Currently, more than sixty peptide drugs have been approved and are on the market for the treatment of cancers and infective and inflammatory diseases [7].

### 2.2. Membrane Permeability of Peptides 

Molecules move across the cell membrane through three pathways of transport include transcellular diffusion, active carrier-mediated transportation, and transcytosis. Molecules that can readily cross cell membranes are useful in modulating intracellular targets. In the pharmaceutical industry, peptides are generally not considered as good drug candidates against intracellular targets due to their limited cell membrane permeability. However, from the literature precedents, we can find that peptides have more space for subtle adjustment of the conformation to improve cell membrane permeability, comparing to small molecule drugs and antibodies. Macrocyclization and backbone *N*-methylation have shown their capacity to accelerate the peptides and peptidomimetics to cross the cell membrane. 

Macrocyclization and *N*-methylation on backbone amide can make changes to steric constraints, the number of hydrogen bond donors (HBDs), and molecular conformations [12]. The changes in physicochemical properties of molecules may have a bearing on membrane penetration. Studies on the relationship between physicochemical properties (e.g., polarity, lipophilicity, and octanol-water partition coefficient) and experimental permeability data can help building guidelines to filter out problematic molecules at the early stages of drug discovery. The “outer limit“ in the cyclic peptide space can be delineated by different molecular properties values (e.g., molecular weight, cLogP, number of hydrogen bond donor, number of hydrogen bond acceptor, polar surface area, and number of rotatable bonds) [13,14,15,16,17,18,19]. Related tactics often operate synergistically; it is hard to identify the individual influence of each property. Computational modeling, on the other hand, can provide valuable insights into the fundamental physics of membrane penetration. Computational methods for accurate prediction of cell permeability remain challenging, but recent developments are promising for the future. 

### 2.3. Advantages of Cyclic Peptides and Peptidomimetics on Cell Permeability 

**Figure 1:**
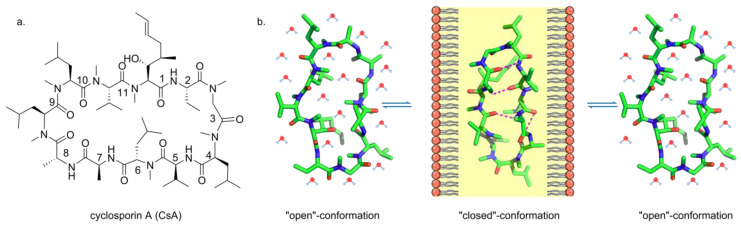
Structure of cyclosporin A (**a**) and mechanisms of cell permeability (**b**).

Macrocycles usually demonstrate more drug-like physicochemical properties that offer the possibility of accessing intracellular proteins, including those involved in PPIs [20]. An interesting and archetypal example is cyclosporin A (CsA). As a cyclic undecapeptide, CsA was found to have high permeability [21,22]. CsA was approved as an immunosuppressant agent in 1997, and it was found to have passive permeability and displayed satisfied oral bioavailability (*F* = 29%). As a ‘beyond-Ro5′ (bRo5) molecule, the mechanism behind CsA’s membrane permeability has been well studied. Structure conformation analysis has revealed that CsA passes membranes by switching between “open-close” conformations [23,24,25]. In apolar solvent, e.g., chloroform, CsA exists as a closed conformation, four intramolecular hydrogen bonds (IMHBs) were formed between Val^5^NH−Abu^2^CO, Abu^2^NH−Val^5^CO, Ala^7^NH−MeVal^11^CO, and Ala^8^NH−MeLeu^6^CO (Figure 1b, IMHBs in purple), the amide bond at MeLeu9−MeLeu^10^ has a *cis*-conformation. Within this elongated sharp IMHB, polar groups of CsA are shielded from the solvent, forming the so-called apolar “closed”-conformation. In contrast, the “opened”-conformation is formed in water (characterized as an aqueous complex that bounds with cyclophilin), with all above IMHBs are broken and releasing the polar groups outward to enhance the interaction with solvents and the protein substrates. Although both conformations share a similar antiparallel β-sheet structure around residues 7–11, the amide bone at MeLeu^9^−MeLeu^10^ changes from a cis to a trans conformation and IMBH related amide protons bonded to cyclophilin instead of forming IMNHs in this polar “open”-conformation. The ability to switch from polar to apolar conformations is crucial for CsA to possess high permeability and good bioavailability, which also opens a window for medicinal chemists to explore the possibility of adjusting the membrane permeability of peptides and peptidomimetics via conformational tuning through chemical modification of the backbone of some macrocyclic peptides.

**Figure 2:**
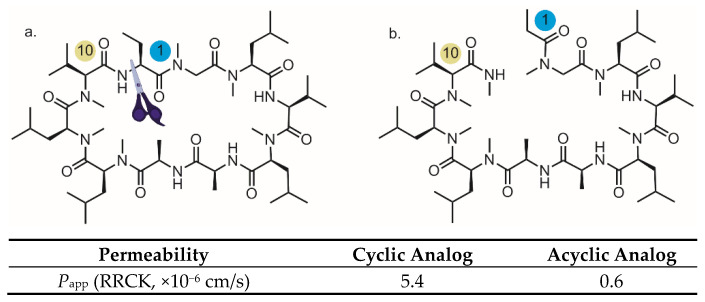
Cyclic CsA analog (**a**) vs. the acyclic analog (**b**).

Cyclization is the most widely applied modification to modulate peptide conformation, aiming to improve their pharmaceutical properties. Cyclization of a linear peptide may reduce the number of intermolecular hydrogen bonds, adjust the lipophilicity, and reduce the hydrodynamic size, thus leading to an increased membrane permeability comparing to its primary linear peptide [26,27,28,29,30,31,32,33,34]. Impacts of cyclization on the pharmacokinetic properties of CsA analogs had been well studied [35]. Pfizer’s report disclosed a novel cyclic CsA analog (Figure 2a) that showed virtually identical *P*_app_ values to CsA in Ralph Russ canine kidney (RRCK) cells. To gain an insight into the cyclization effect on CsA analog’s physicochemical profiles, an acyclic analog (Figure 2b) was also prepared. To minimize the influences of structural changes on overall physicochemical profiles, a linear peptide was designed and synthesized, which had equal total carbon, oxygen, and nitrogen atoms. The macrocycle was disconnected at the amide bond between Val^11^ and Abu^1^, and Abu^1^ was replaced with a propionic side-chain, and the Val^11^ carboxylic acid was masked as *N*-methyl amide. Significantly reduced permeability of the acyclic analog was observed in RRCK cells, which clearly showed the impact of cyclization on the membrane and cell permeability of peptides with identical sequences but different conformations [35]. Many more examples [36,37,38] have been reported that macrocyclic peptides have better pharmacokinetic properties compared to their linear counterparts, which have huge potential for drug discovery.

## 3. Backbone *N*-Methylation: Pivotal Roles to Improve the Permeability for Cyclic Peptides and Peptidomimetics

Macrocyclic skeletons usually have advantages over their linear counterparts and have attracted lots of attention from academics and industry. In the field of cyclic peptides drug discovery, several kinds of structural modifications have been developed to improve bioavailability, which has been well documented in other review articles [39].

α-Carbon modifications, *N*-methylation, and isosteres of the amide bond are frequently used methods in the field of structural modifications of cyclic peptides. Of these methods, the backbone *N*-methylation is a preferable strategy to fine-tune the structural conformation of cyclic peptides [40,41]. Regiospecific *N*-methylation of backbone amide, (i) introduces additional steric constraints, (ii) selectively blocks one hydrogen bond of the original amide NH, and (iii) lowers the transforming energy of the amide bond from a cis-configuration to a trans-form or vice versa. *N*-methylation of amides enables the molecule to adopt cis conformations far more readily than standard unmethylated ones. Selective backbone amide *N*-methylations allow peptide macrocycles to automatically adopt specific conformations according to different circumstances in vitro or in vivo, which play important roles in conserving the target molecules with improved membrane permeability. As being discussed in the case of CsA, with high levels of backbone amide *N*-methylations, 7 of 11 amides in the backbone were methylated, *N*-methylations reduced the number of hydrogen bonds, and helped the cyclic peptide to adopt a cis-conformation at MeLeu^9^−MeLeu^10^.

Inspired by these natural products that are inherently methylated, *N*-methylation of backbone amides has become an important method to improve the drugability and pharmacokinetics of cyclic peptides as drug candidates [41,42]. In this review, we provide an update on the latest reports in this field (please refer to refs [12,39,41,42] for previous reviews on *N*-methylation of peptide). This paper will first introduce the methods that are useful for the construction of *N*-methylated amides on the backbone of cyclic peptides and peptidomimetics, followed by a discussion on the impacts of *N*-methylation of backbone amides on membrane permeabilities of the desired substrates.

### 3.1. Chemical Synthesis of N-Methylated Cyclic Peptides

Methods elected to introduce *N*-methyl groups are usually determined by which strategy will be applied to gain the final macrocyclic peptides or peptidomimetics. Solution-phase synthesis of peptides usually employ *N*-methylated amino acids (NMAAs) as building blocks; these NMAAs are prepared in advance using different synthetic methods, while for the solid-phase synthesis of peptides, it is more convenient to install a methyl group on nitrogen of the amino acid moiety during the elongation of the peptide chain, which is more region-specific and has high efficiency.

#### 3.1.1. Preparation of N-Methyl Amino Acids (NMAAs) as Building Blocks for Solution-Phase Synthesis of Peptides

**Scheme 1:**
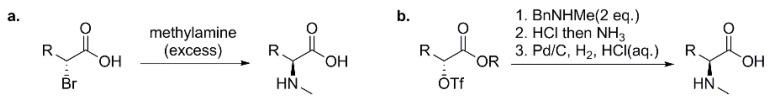
*N*-Methylation via S_N_2 substitution of α-bromo acid (**a**) or α-triflate esters (**b**).

Chemists have established several reliable methods for the synthesis of NMAAs [43,44]. S_N_2 displacement of α-bromo acids was first performed by Fischer and Mechel (Scheme 1a), providing the free NMAAs directly without additional protection and deprotection procedures [43]. When optically active, α-bromo acids were treated with excess methylamine, and the stereogenic center was reversed (for example, (*R*)-2-bromopropionic could easily be transformed to *N*-methyl-L-Ala). The main drawbacks of this method are obvious; it usually suffers from low yields and racemization. Although an alternative approach was reported by the Effenberger group (Scheme 1b), employing triflate as the leaving group made the reaction conditions milder and improved the optical purity of NMAAs [45], and it complicated the preparation of starting materials, which limited its applications in practical synthesis.

**Scheme 2:**
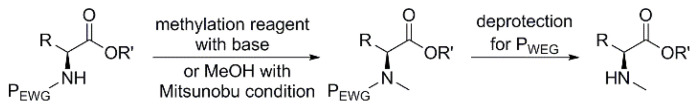
Preparing *N*-methyl amino acid by alkylation on a protected amine.

Selective *N*-alkylation of properly protected amino acids is one of the most used preparation technologies for NMAAs (Scheme 2). Sequentially protecting the amino group with electron-withdrawing groups (P_EWGs_, e.g., sulfonamides, carbamates, and amides), and treatment the intermediate with methyl iodide, diazomethane, trimethyloxonium tetrafluoroborate, and dimethyl sulfate with or without a combination of base or acid scavengers, the corresponding NMAAs can be prepared in good yields with the alpha-stereogenic center intact [43]. P_EWGs_ are essential for these methodologies, which can enhance the acidity of the NH group and make it possible that the subsequent *N*-methylation happens in the presence of very mild bases or even acid scavengers. Methanol can also serve as a methyl group source when the Mitsunobu reaction condition is employed [46,47].

**Scheme 3:**
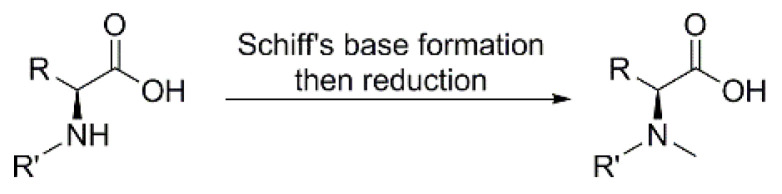
Preparing *N*-methyl amino acid by Schiff’s base reduction.

Reductive methylations can be categorized into two methods: (i) reduction of imine (Schiff base) and (ii) reduction of oxazolidine. Schiff’s base reduction can be performed using many kinds of reagents, for examples, borohydrides (sodium cyanoborohydride and triacetoxyborohydride), formic acid (Leuckart reaction), borane, or transition metal-mediated reduction, of the intermediate formed by the condensation of formaldehyde with an amine group of amino acids (Scheme 3) [44,48]. 

**Scheme 4:**
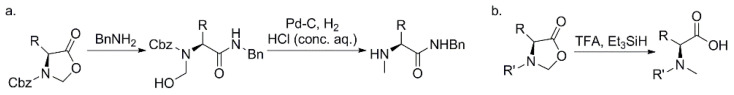
*N*-methylation via reductive ring opening of oxazolidin-5-one: palladium-catalyzed hydrogenation (**a**) and reduction with triethylsilane/TFA (**b**).

Ben-Ishai first noticed that oxazolidin-5-ones were susceptible to nucleophilic attack (Scheme 4a) [49]; the *N*-methylol amide could be easily transformed to *N*-methylated amide via palladium-catalyzed hydrogenation [49] or reduction with triethylsilane/TFA combination [50]. Freidinger and co-workers further optimized this method, directly reductive ring-opening to furnish the protected *N*-methylated amino acids was achieved in one-pot and single step of reaction in the presence of triethylsilane/trifluoroacetic acid (TFA) (Scheme 4b) [51]. This technique was applicable to the synthesis of *N*-Fmoc- or *N*-Cbz- protected NMAAs [52,53] and has already been extended for the preparation of *N*-Boc-protected NMAAs in neutral reaction conditions by utilizing a mild hydrogenation procedure [54]. 

With the expanding of toolkits, many kinds of protected NMAAs are available; the *N*-protective groups range from oNs, Boc, Cbz to Fmoc, etc., while the starting materials cover nearly all proteinogenic amino acids. These NMAAs have been widely used in solution-phase and solid-phase synthesis of *N*-methylated peptides; most of these kinds of building blocks are even commercially available today.

#### 3.1.2. Regio-Specific N-Methylation for Solid-Phase Synthesis of Peptides

**Scheme 5:**
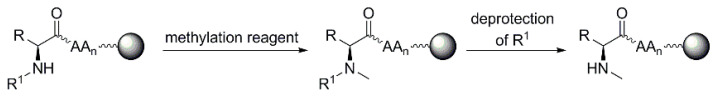
*N*-methylation of amino acid and peptide chain in SPPS.

Solid-phase peptide synthesis (SPPS) is a widely used technique for peptide synthesis. To incorporate NMAAs in SPPS usually suffers from steric hindrance, leading to low efficacy of peptide coupling and difficulties for purification. To install the *N*-methyl group on a specific unit of an amino acid after it has been attached to the peptide chain using the classical coupling method for SPPS, would be a good choice. Fukuyama and co-workers introduced a versatile and efficient protective group for primary amine, the o- or p-nitrobenzene sulfonyl (nosyl, o-, and p-NBS-Cl) [55], these groups are easy to introduce as *N*-protecting groups for amino acids, and due to their strong electron-withdrawing features, they are stable and suitable for *N*-methylation reaction in SPPS under very mild reaction conditions, for example, the Mitsunobu reaction. Miller and Scanlan [56,57] extended this method for *N*-methylation of specified amino acids in SPPS, which are wildly used by many research groups (Scheme 5) [46]. The procedure was further optimized by the Kessler group with a significant reduction in time and cost [58].

Removal of these nosyl groups usually need strong nucleophiles; the Locky group reported *N*-trifluoroacetamide (Tfa) as an *N*-protecting group and that it was also applicable for regio-selective *N*-methylation in SPPS, [59], and unlike nosyl groups, the Tfa group can be reliably cleavaged using sodium borohydride, which is completely orthogonal to most standard protecting groups employed in SPPS.

### 3.2. Backbone N-Methylation in the Discovery of Permeable Cyclic Peptide/Peptidomimetic

*N*-methylation, as a chemical modification, could be utilized in the design of peptides to improve their drug-like properties. In recent years, several investigations have been reported about the influences of backbone *N*-methylation on the permeability of cyclic peptides. 

#### 3.2.1. Studies on Methylated Analogs of Sanguinamide A

**Figure 3:**
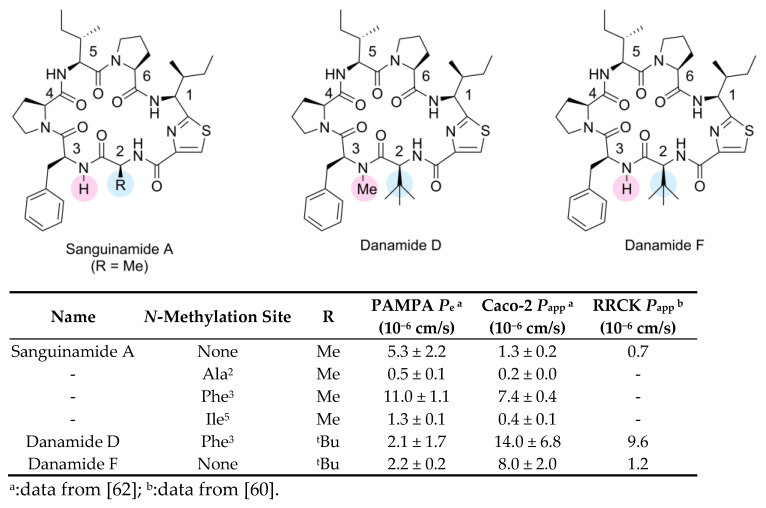
Methylated analogs of Sanguinamide A.

Sanguinamide A was isolated from *Hexabranchus sanguineus* as a novel thiazole-containing macrocyclic heptapeptide (cyclo- [Ile(Thz)-Ala-Phe-Pro-Ile-Pro]). Many efforts have been devoted to the synthesis of Sanguinamide A and related analogs [60,61,62]. Sanguinamide A has also been a good molecular template for the study of relationships between *N*-methylation on its backbone amides and the changes of conformation and permeability (Figure 3). 

The first total synthesis of Sanguinamide A was reported in 2012 by the Fairlie group [61]. The total chemical synthesis revised the cis,cis-amide bonds to *cis*,*trans*-amide bonds in this macrocyclic peptide, and some interesting conformational information were found in NMR studies. Two intramolecular hydrogen bonds, Ala^2^NH-COIle^5^ and Ile^5^NH-COAla^2^, were strong enough to keep the macrocycle locked in one stable conformation, which left the hydrophobic side-chains of aminol acids outward of the macrocycle, shielding the other hydrophilic amide groups interiorly from water. Supported by H-D exchange experiments, amide NH protons in Ala^2^ and Phe^3^ were found more solvent-exposed than others [60]. A series of analogs of Sanguinamide A were synthesized from commercially available Fmoc-*N*-methyl-amino acid by solid-phase synthesis, and a systemically study on the permeability of Sanguinamide A and its analogs were also reported [60,61]. Compared to Sanguinamide A, analogs with the removal of the thiazole moiety (cyclo-(Ile-Ala-Ala-Phe-Pro-Ile-Pro)) or the introduction of a bulky tertiary butyl glycine at position 2 (Danamide F) did not significantly affect their permeability (RRCK, *P*_app_ = 0.6 × 10^−6^ cm/s; 1.2 × 10^−6^ cm/s); however, an analog with *N*-methylation on Phe^3^ (Danamide D) significantly improved the RRCK cell membrane permeability to 9.6 × 10^−6^ cm/s. 

The Lokey group reported more results on the impact of backbone *N*-methylation of the Sanguinamide A scaffold, [62] they noted that the effect of backbone *N*-methylation on permeability was highly position-dependent, the *N*-methylation at the Ala^2^-NH and Ile^5^-NH broke the transannular hydrogen bonds and significantly reduced both parallel artificial membrane permeability (PAMPA) and Caco-2 permeability, when *N*-methylation was applied to Phe^3^-NH, it resulted in dramatic improvements to permeability. 

#### 3.2.2. PAMPA Permeability of N-Methylated LB51 Analogs

**Figure 4:**
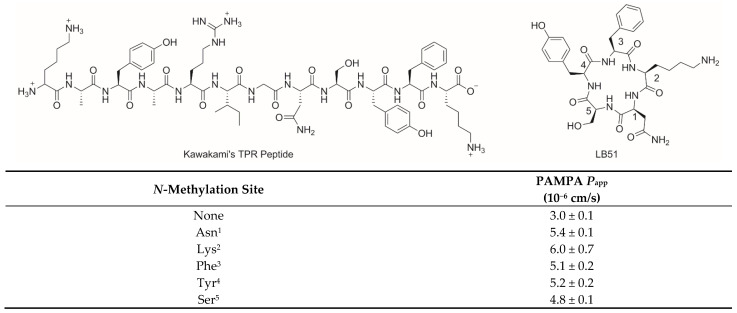
PAMPA permeability of *N*-methylated LB51 analogs.

TPR2A, one of three TPR domains in Heat shock organizing protein (HOP), plays an important role in the interactions of the MEEVD region with Hsp90 [63,64,65]. The TPR peptide was discovered by Kawakami and co-workers as a mimic (Figure 4) of the TPR2A domain, which could disrupt Hsp90-HOP binding [66]. Aiming for a promising lead compound, several truncated linear peptides and their cyclic variants were synthesized. A cyclic analog, LB51 showed promising activity on Hsp90b-Cyp40 binding inhibition with an IC_50_ value at low micromolar levels [67]. Owing to the presence of four highly polar side chains in this cyclic pentapeptide, poor cell permeability became the greatest challenge for further development. Backbone *N*-methylation was elected to archieve some breakthroughs in membrane permeability. The McAlpine group reported systemic studies on backbone amide *N*-methylation at each amino acid, which produced five analogs of LB51 [68]. The *N*-methylated analogs were archived by *N*-methylating amino acids on solid-phase with the Miller and Scanlan approach [56,57]. The resin-bound amino acid or peptide was methylated with the Mitsonobu reaction [46,47]. All of these analogs showed significantly improved membrane permeability over the original lead molecule.

#### 3.2.3. Studies on N-Methylated Analogs of Cyclo(-Pro-Phe-D-Trp-Lys-Thr-Phe-)

**Figure 5:**
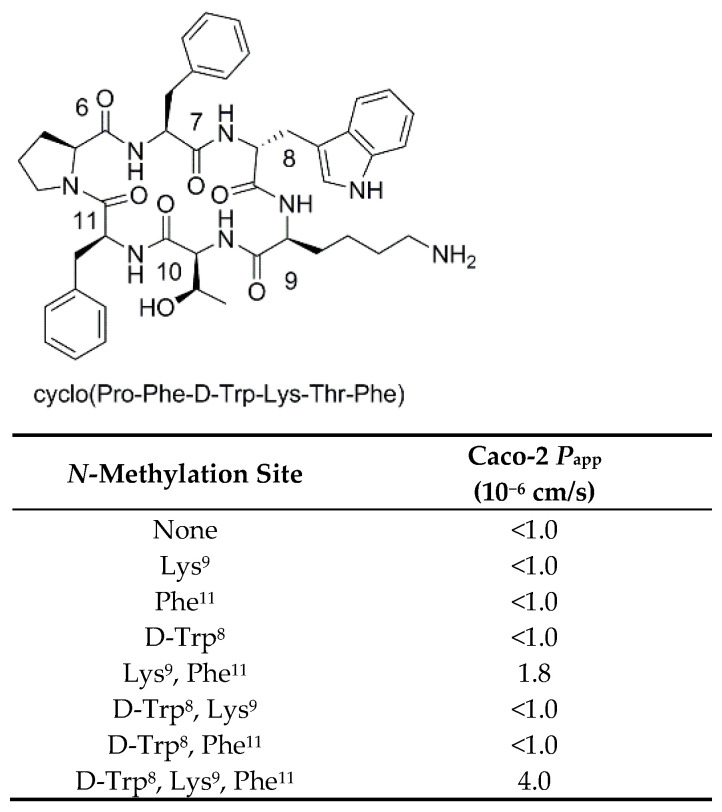
Membrane permeability of methylated analogs of cyclo(-Pro-Phe-D-Trp-Lys-Thr-Phe-).

Cyclic hexapeptide cyclo(-Pro-Phe-D-Trp-Lys-Thr-Phe-) is a synthetic somatostatin mimic that has selective inhibition activity towards sst2 and sst5 subtypes of somatostatin receptor. Synthetic somatostatin analogs have been wildly used in the diagnosis and treatment of somatotropinomas, thyrotropinomas, and functioning and non-functioning gastroenteropancreatic neuroendocrine tumors. However, parenteral drug administration is the only dosing method because of its low oral bioavailability. Kessler, Hoffman, and co-workers discovered several *N*-methylated analogs of cyclo(Pro-Phe-D-Trp-Lys-Thr-Phe) with better intestinal permeability and enzymatic stability that would be orally available (Figure 5) [69]. A library of *N*-methylated peptides was synthesized on a solid support (linear peptides) and cyclized in solution. The Fmoc-MePhe-OH building block was prepared following the procedure described by the Freidinger group, while other *N*-methylated amino acids were generated using an optimized Miller and Scanlan approach [56,57,58]. In the library of 30 *N*-methylated peptides, analog with triple-*N*-methylation on D-Trp^8^, Lys^9^, and Phe^11^ showed the highest intestinal permeability (Caco-2, *P*_app_ = 4 × 10^−6^ cm/s). This compound improved oral bioavailability without any loss of its biological activity and selectivity. 

#### 3.2.4. Membrane Permeability of N-Methylated Poly Alanine Cyclic Pentapeptide/hexapeptide

**Figure 6:**
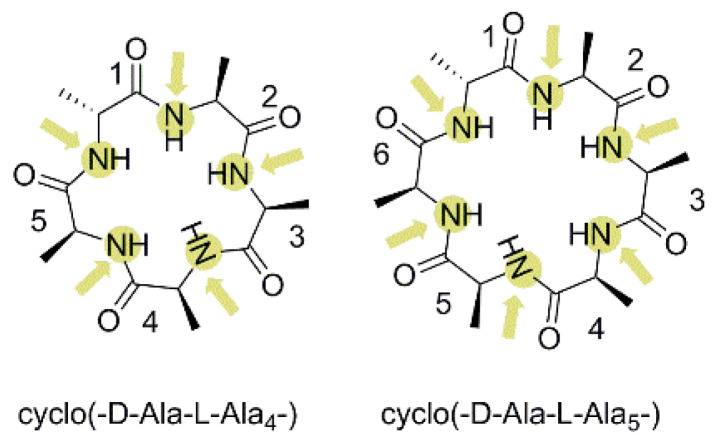
Polyalanine cyclic pentapeptide/hexapeptide.

The Kessler group reported the extensive conformational studies of poly-alanine cyclic peptides, using cyclic poly-alanine peptides as templates, to survey the structural requirements that convey permeability. To clarify how backbone amide *N*-methylation improves cell permeability, it is essential to sort out the impacts of *N*-methylation on cyclic peptide conformation. Derived from the basic sequence cyclo(-D-Ala-L-Ala_4_-), a library with 30 different *N*-methylated cyclic peptides (Figure 6) was generated [70]. *N*-methyl alanine was synthesized in solution using the Freidinger approach [51] and used on solid-phase like a normal amino acid. A conformational study on constrained cyclic pentaalanine peptides indicated that *N*-methylation on cyclic peptides led to high variability in their conformations. A systematic investigation of the relationship between backbone *N*-methylation and intestinal permeability of hexa-Ala peptide was also reported by the Kessler group in 2011 [71]. A polyalanine cyclic hexapeptide library (Figure 6) that varied in the number (1-5 *N*-Me groups) and positions of *N*-methyl groups was synthesized in SPPS and screened for intestinal permeability through a Caco-2 cell monolayer. The cyclic hexa-Ala peptide had low permeability, but 10 out of the 54 *N*-methylated derivatives were found to have a high permeability rate; some of them had similar permeability to that of testosterone (a wildly used passive transcellular permeability marker). These studies clearly showed that alteration of the site and/or numbers of *N*-methylation on the backbone amides of a cyclic peptide could make great impacts on permeability. Further studies from the Kesser group suggested that multiple backbone *N*-methylation could dramatically improve their Caco-2 permeability; there were two preferable conformational templates with high Caco-2 permeability [72]. One template possessed two β-turns of type II along Ala^6^-D-Ala^1^ and Ala^3^-Ala^4^; another one possessed a type-VI β-turn geometry along Ala^4^ and Ala^5^. Mechanistically, carrier-mediated transporters are involved in the improvement of cell permeability [73]. 

#### 3.2.5. Studies on N-Methylated Analogs of Cyclo(-Leu-Leu-Leu-Leu-Pro-Tyr-)

**Figure 7:**
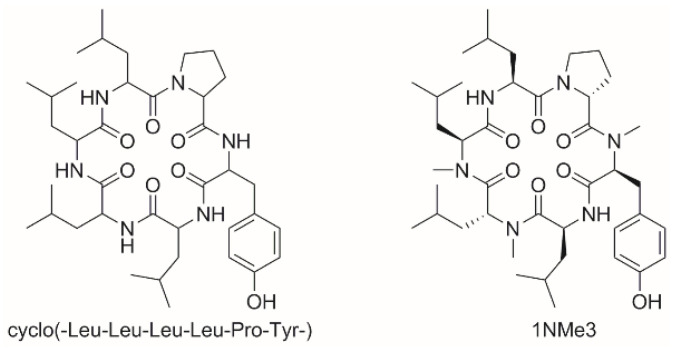
Cyclo(-Leu-Leu-Leu-Leu-Pro-Tyr-) for the discovery of orally bioavailable scaffolds.

Using a selective on-resin *N*-methylation method, [74] the Lokey group created a library of cyclic hexapeptides (Figure 7) with different degrees of *N*-methylation. Passive membrane diffusion rates were tested in the parallel artificial membrane permeability assay. The conclusions happened to coincide with Kessler and other research groups; on the one hand, the intramolecular hydrogen bonds played pivotal roles in conformational control, intramolecular hydrogen bonds would not adversely affect permeability because they could lock the hydrophilic groups inside the macrocycle and leave the hydrophobic side-chains outside the molecule; on the other hand, backbone *N*-methylation increased steric hindrance regio-specifically that could be helpful for the molecules to adopt preferential confirmation with better permeability. Comparing to intramolecular hydrogen bonds, *N*-methylation was more intrinsic as it could be resistant to environmental changes. However, over-methylated substrates did not inherit all of these advantages. Partially and region-specifically *N*-methylated analogs were more permeable than the original peptides and the permethylated analogs. A partially methylated compound (namely 1NMe3) with good cell permeability and microsomal stability was discovered. 1NMe3 showed intravenous absolute oral bioavailability (*F* = 28%, similar to that of CSA) in in vivo pharmacokinetic studies. Advanced studies on the pharmacokinetic features of 1NMe3 will benefit the understanding of molecules with similar structures and attributes. 

#### 3.2.6. Backbone N-Methylation on Modulators for Chemokine Receptor CXCR7

**Figure 8:**
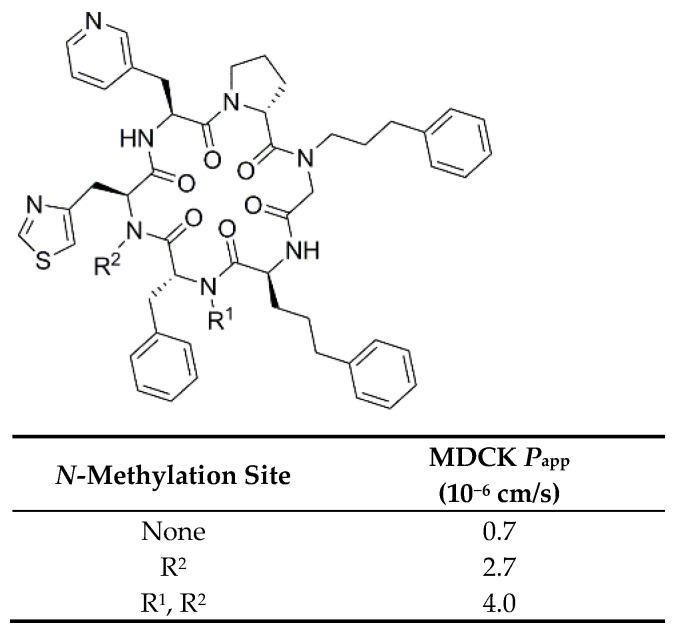
Backbone *N*-methylation to improve permeability.

New knowledge generated from previous studies can be applied in the optimization of other scaffolds. On the development of modulators for chemokine receptor CXCR7, strategies, such as peptoid variations, side-chain replacement, and backbone *N*-methylation, have been applied to gain cyclic peptides with improved binding affinity and passive permeability [75]. Backbone *N*-methylation at R^1^, R^2^ has been shown to have a beneficial effect on permeability in these scaffolds (Figure 8). 

#### 3.2.7. Influence of N-Methylation on Permeability of Semipeptide Macrocycles

**Figure 9:**
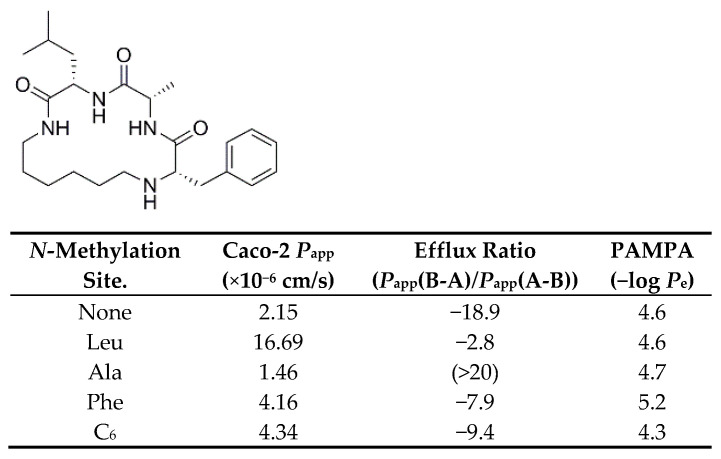
Influence of *N*-methylation on the permeability of semipeptidic macrocycles.

Semipeptidic macrocycles now play important roles in drug development. However, different from macrocyclic peptides, backbone *N*-methylation on semipeptide macrocycles may not produce similar results. The Marsaultp group provided a detailed analysis of the structure-permeability relationship of semipeptidic macrocycles [76]. From easily prepared Fmoc-(*N*-Me)-Phe-OH and other commercially available Fmoc-*N*-methyl-amino acids, semipeptidic macrocycles analogs with different *N*-methylation state were synthesized. Compared to a non-*N*-methylated scaffold, *N*-methylation on Leu, Phe, and alkyl linker C_6_ had positive impacts on efflux ratio, while other modifications showed opposite potentials. As illustrated in Figure 9, *N*-methylation changed the efflux ratio and cellular permeability on Caco-2 cells in a site-specific manner but had little influence on passive permeability. The unique alkyl linker in semipeptides imparted higher flexibility and could not accommodate the strong transannular IMHBs. Taken all these factors together, the behaviors of backbone *N*-methylation on semipeptide macrocycles displayed significant differences from that of peptide macrocycles. *N*-methylations on this semipeptide affected IMHB patterns but showed different results of cell permeability. The influences of the ring size, sequence, and expanding side-chain diversity on permeability were also studied in this report.

#### 3.2.8. Membrane Permeability of Hirsutellide A and Its Desmethyl Analog

**Figure 10:**
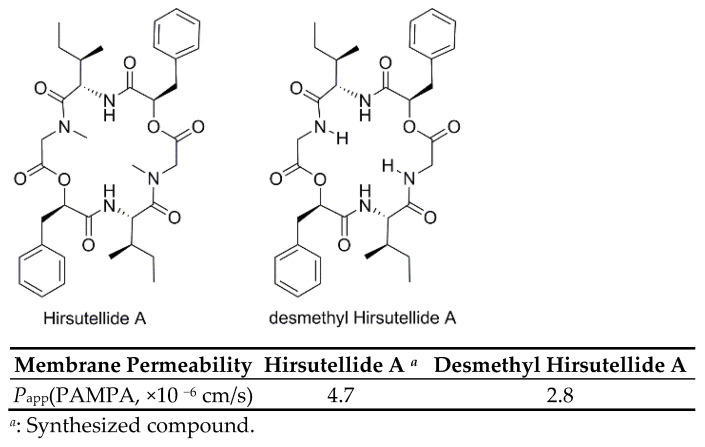
Membrane permeability of Hirsutellide A and desmethyl analog.

Hirsutellide A (Figure 10) is an 18-membered cyclic hexadepsipeptide with in vitro antimycobacterial (*M. tuberculosis* H37Ra, MIC = 6-12 μg/mL) and antiplasmodial (*Plasmodium falciparum*, IC_50_ = 2.8 μg/mL) activities [77,78]. To verify its structural configuration and the structure-activity relationship, series of depsipeptide and peptide analogs of hirsutellide A were prepared by the Imming group [79]. To evaluate the role of *N*-methylation on retaining biological activities and ADME profiles, a demethylation analog (Figure 10) of the *N*-methyl sarcosine was prepared. The demethylated analog showed a positive effect on the passive artificial membrane permeability but with reduced antiplasmodial activity and plasma stability.

#### 3.2.9. Influence of Backbone *N*-Methylation on Permeability of Lariat Peptides

**Figure 11:**
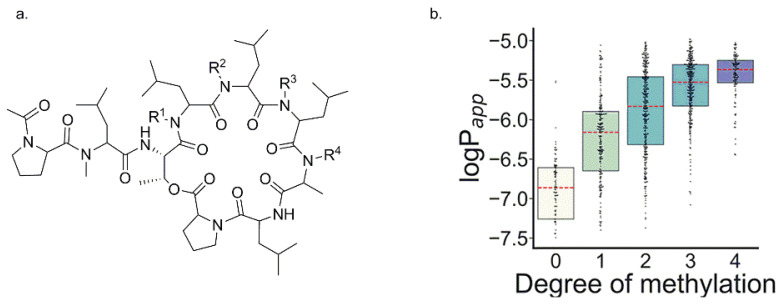
Structure (**a**) and permeability (**b**) of lariat peptides. Adapted with permission from the authors [80] Copyright (2021): American Chemical Society.

The split-pool bead method provides access to large and well-diversified chemical libraries. The Lokey group generated a library of novel lariat peptide scaffolds (Figure 11) with molecular weights around 1000 Da [80]. The library with over four thousand compounds was screened for permeability. Many lariats were surprisingly permeable, comparable to many known orally bioavailable drugs. Relationships between structure and permeability for lariats were well summarized, with extensive variation in backbone *N*-methylation, stereochemistry, and ring topology. On the aspect of *N*-methylation, Fmoc-*N*-methylated amino acids as building blocks were introduced in the solid-phase peptide synthesis to produced libraries with different degrees of methylation. Comprehensive analysis showed that compounds with more *N*-Me groups were more permeable, but *N*-methylation performs differently in different positions.

#### 3.2.10. Backbone N-Methylation of Hexa-, Hepta- and Octo-Thioether-Containing Cyclic Peptides

**Figure 12:**
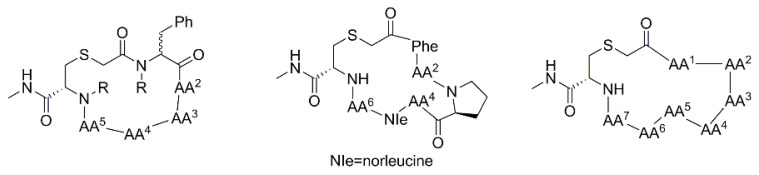
Structures of hexa-, hepta-, and octo-thioether-containing cyclic peptides.

The Monovich group reported an in silico optimization guided discovery of permeable and orally exposed cyclic peptidomimetics [81]. Specific thioether-containing macrocycles (Figure 12) were chosen as the parent skeleton for the investigation. Target macrocycles with backbone *N*-methylations are synthesized in the solid phase from Fmoc-*N*-methylated amino acids. The impact of the *N*-methylation pattern on the permeability was first carried on the thioether-containing hexapeptide macrocycles skeleton. For molecules that had different degrees and positions of *N*-methylation, one with double methylations on peptide backbone at AA^4^R or AA^3^AA^5^ showed high passive permeability (PAMPA, log *P*_app_ = −4.3). Others verified significantly on the permeability property with differences in the numbers or locations of *N*-methylation. Several facts could be contributed to passive permeability, and physical models of passive membrane permeation were evaluated on the cyclic hexapeptide system. In advance of chemical synthesis, a combination of 3D physics-based predictors, such as ΔG^*^_transfer_ and a number of solvent-exposed NHs with conformational analysis, should facilitate the identification of permeable and orally exposed cyclic peptidomimetics. This strategy was further applied to the hepta- and octo- thioether-containing macrocyclic systems.

## 4. Summary and Perspectives

Today, more than 60 peptide drugs have been approved; more than half of them are cyclic peptides. Peptides have a three-dimensional configuration that can adopt particular conformations for binding to proteins, which are well suited to interact with larger PPI contact surface areas on target proteins. However, low cell permeability is a major challenge in the development of peptide therapeutics. In recent years, peptide-focused structural studies on macrocycle structure have pointed out the promising of cyclic peptide/peptidomimetic. Amides are unquestionably one of the most intrinsic functional groups in peptides, and *N*-methylation is a wildly used tool to manipulate permeability. Backbone *N*-methylation permits the adjustment of the molecule’s conformational space. Several pathways are involved in the drug absorption; the relative importance of each *N*-methylation to total permeation is likely to differ with intrinsic properties of cyclic peptide/peptidomimetic. The current understanding of the relationship between cyclic peptide structure and its permeability could not provide a quantitative comparison across macrocyclic peptide/peptidomimetic systems; we hope these problems can be solved in future studies.

Broadly speaking, the current topic may be extended to small proteins such as affibodies [82,83,84] and nanobodies [85,86]. Affibodies are composed of 50−60 amino acids, with molecule weight ranging between 6−7 kDa, while nanobodies are barely higher than 15 kDa. Owing to their small size, they have congenital advantages to penetrate membranes upon the conjugation with cell-penetrating peptides (CPPs) [87,88,89] or by resurfacing with polycationic residues [90]. Like the above strategies, increasing the net charge by esterifying the surface carboxylic acids could also increase the cell-permeability of GFP [91], which might be applicable to these small proteins. However, it is yet to be investigated whether backbone *N*-methylation, the topic of this review, could be applied to manipulate the permeability of affibodies or nanobodies as efficiently as for macrocyclic peptides.

## Data Availability

Not applicable.
